# Gelation Time of Network-Forming Polymer Solutions with Reversible Cross-Link Junctions of Variable Multiplicity

**DOI:** 10.3390/gels9050379

**Published:** 2023-05-04

**Authors:** Fumihiko Tanaka

**Affiliations:** Department of Polymer Chemistry, Graduate School of Engineering, Kyoto University, Katsura, Kyoto 615-8510, Japan; ftanaka@kmj.biglobe.ne.jp

**Keywords:** thermoreversible gelation, gelation time, relaxation time, multiple cross-links, stepwise association

## Abstract

The gelation time $t_{g}$ necessary for a solution of functional (associating) molecules to reach its gel point after a temperature jump, or a sudden concentration change, is theoretically calculated on the basis of the kinetic equation for the stepwise cross-linking reaction as a function of the concentration, temperature, functionality *f* of the molecules, and multiplicity *k* of the cross-link junctions. It is shown that quite generally $t_{g}$ can be decomposed into the product of the relaxation time $t_{R}$ and a thermodynamic factor *Q*. They are functions of a single scaled concentration x≡λ(T)ϕ, where λ(T) is the association constant and ϕ is the concentration. Therefore, the superposition principle holds with λ(T) as a shift factor of the concentration. Additionally, they all depend on the rate constants of the cross-link reaction, and hence it is possible to estimate these microscopic parameters from macroscopic measurements of $t_{g}$. The thermodynamic factor *Q* is shown to depend on the quench depth. It generates a singularity of logarithmic divergence as the temperature (concentration) approaches the equilibrium gel point, while the relaxation time $t_{R}$ changes continuously across it. Gelation time $t_{g}$ obeys a power law $t_{g}^{-1}∼x^{n}$ in the high concentration region, whose power index *n* is related to the multiplicity of the cross-links. The retardation effect on the gelation time due to the reversibility of the cross-linking is explicitly calculated for some specific models of cross-linking to find the rate-controlling steps in order for the minimization of the gelation time to be easier in the gel processing. For a micellar cross-linking covering a wide range of the multiplicity, as seen in hydrophobically-modified water-soluble polymers, $t_{R}$ is shown to obey a formula similar to the Aniansson–Wall law.

## 1. Introduction

Thermoreversible gelation in solutions of polymers, as well as of low molecular weight molecules, has been attracting researchers’ interest [1,2,3,4,5,6,7]. Many examples of the phase diagrams with sol–gel transition lines have been reported in the literature. Some original studies, reviews and conceptual works have appeared with relation to responsive gels [8,9,10,11], hydrogels for biomedical applications [6,7,12], and hydrogen-bonding supramolecular gelators [13,14,15]. However, the kinetic process of cross-linking reaction to reach the gel point, in particular the gelation time, has not been clarified yet. Here, the gelation time $t_{g}$ is defined by the time necessary for the network-forming solutions of functional molecules to reach their gel points after a cross-linking reaction is started. It is related to the relaxation time $t_{R}$ for the solution to go back to their thermal equilibrium state, but must be strictly distinguished from each other because the former goes to infinity on the equilibrium sol–gel transition line while the latter remains continuous across it.

Fundamental investigation of polymer cross-linking reactions as a function of time is very important to obtain better understanding of the gelation mechanism for controlling the production process and final performance of gels. In particular, acceleration of the gelation time in the most efficient way under restricted conditions is critical for the application of gels to biomedical technology, the food industry, the adhesion industry, etc.

Gelation time is also very important from a scientific viewpoint. It includes microscopic information on the kinetics of cross-linking reaction. By the macroscopic measurements of the gel point by rheology, we can speculate on the microscopic structure and formation process of the network junctions, in particular the rate-controlling steps in cross-linking.

The gelation time critically depends on the thermodynamic conditions under which the cross-linking reaction proceeds, such as temperature, concentration of the functional molecules, their mixing ratio if there are more than one component, amount of the cross-linkers if any, pH, etc. The purpose of the present study is to clarify the difference between $t_{g}$ and $t_{R}$, and find them as functions of the functionality of the molecules, multiplicity of the junctions, and the system parameters such as concentration and temperature by focusing on their relations to the rate constants of reaction in the kinetic equation.

Experimental data of the gelation time $t_{g}$ in the literature obtained by the rheological measurements, scattering, calorimetry, etc. are usually plotted against the temperature and/or polymer concentration to find the activation energy in the rate constant and the power index of the concentration. For instance, Ohkura et al. [16,17] reported the data on the gelation time of poly(vinyl alcohol) (PVA) in a mixed solvent of water and dimethylsulphoxide. They measured the time by a ball-drop method in the temperature quenching experiments for various polymer concentrations. The results were summarized in the form

(1) $$t_{g}^{-1}=A\left(T\right){\left(\Delta \phi \right)}^{n}$$

where Δϕ is the concentration of PVA measured from the overlap concentration. The critical concentration at which $t_{g}$ becomes infinite was found to be independent of the temperature. (The log–log plot of this equation is referred to as *Oakenfull plot* in p.80 of the reference [2].) The exponent *n* was found to be n=2 under the assumption that the gel point is given by the overlap concentration. Because the cross-link junctions are formed by micro-crystallites, the temperature factor was found to agree with the nucleation rate

(2) $$A\left(T\right)≃e^{-B/T{\left(\Delta T\right)}^{2}}$$

where *B* is a temperature-independent constant.

Investigation was done by Mal et al. [18] on the solutions of crystalline polymer poly(vinylidene fluoride) (PVF${}_{2}$) in two different solvents. The results were plotted in the form of (1). The critical concentration depends on the temperature, from which they constructed the sol–gel transition line for three samples. The gel point depends on the nature of the solvent; gelation is easier in a poor solvent. The exponent of concentration was found to be n≃ 0.45–0.60, much smaller than the value for PVA.

Hong et al. [19] studied the same polymer PVF${}_{2}$ in tetra(ethylene glycol) dimethyl ether. They confirmed that the temperature factor takes a crystalline nucleation form, but found a crossover in the power law from n=2.17 to n=1.18 as the critical gel point is approached. Based on these data, a new model of gel formation in crystalline polymers was proposed.

A similar study was done by Tobitani et al. [20,21] for heat-setting gels of protein, bovine serum albumin (BSA) and beta-lactoglobulin (β-Lg), in order to construct the equilibrium sol–gel transition curves by the measurement of dynamic-mechanical moduli. Data were again summarized in the form of (1). Because the gelation is accompanied by protein denaturation, the temperature factor A(T) took an activation type. They also found the exponent of the concentration to be much higher than two. To incorporate these results, they proposed a new model of the gelation time by considering *multiple reaction* of functional groups in the cross-link junctions [22,23,24]. We will refer to their idea again in the section treating multiple cross-links. The activation type temperature dependence of the gelation time has been reported for some other gel-forming polymers, such as polyurethane dispersion [25], silica alkoxides [26,27], etc.

Investigation on the gelation time was reported in the literature for the binary cross-linking in the mixture of functional molecules [28,29,30,31,32]. The main problem of binary gels is to find the optimal mixing ratio for minimizing the gelation time, so that plot of $t_{g}$ against the mixing ratio for different quenching temperatures is fundamental.

From the theoretical side, there have been accumulating studies based on the kinetic equations for the cross-linking reaction of functional groups in the classical Flory–Stockmayer pictures of gelation. For instance, Stockmayer [33,34] derived the rate equation of *irreversible* gelation for the reactivity (conversion) α as a function of time, and found its value at the gel point.

Later, Ziff et al. [35,36,37,38] extended Stockmayer’s model on the basis of a more general kinetic equation of cluster formation to include the time development of reactivity in the post-gel region. They treated not only unary cross-linking (self-reaction), but also binary cross-linking (copolymerization) in their series of papers [36]. The scaling and universality of gel formation was also discussed [38]. Dongen et al. [39] considered *reversibility* of the cross-linking reactions, and studied retardation effects due to the existence of reverse reaction. However, unfortunately, the effects of temperature and concentration were not explicitly considered in these studies.

In spite of this diversity in experimental and theoretical studies, there has been no attempt to construct a unified picture of the gelation time of polymer solutions by starting from the fundamental kinetics of the cross-linking reaction. In this paper, we focus on the reversibility and variable cross-link multiplicity of a stepwise rate equation for the cross-linking reaction. Solving the rate equation for the probability $p_{k}\left(t\right)$ of cross-linking with multiplicity *k*, we derive the gelation time $t_{g}$ as a function of the polymer concentration and temperature. In contrast to (1), it includes a temperature–concentration cross-term. The results are expressed in the common form $t_{g}=t_{R}Q$ in terms of the relaxation time $t_{R}$ and a thermodynamic factor *Q*. The latter shows a singularity near the equilibrium sol–gel transition line. We investigate the retardation effect due to the reverse reaction (dissociation) and the effect of variable multiplicity of the junction. The gelation time for the reversible binary cross-linking will be reported in our forthcoming paper.

## 2. Theoretical Method

The model solution we consider is a polymer solution in which the number *N* of reactive (associative) molecules (denoted as R{A${}_{f}$}) with degree of polymerization *n* are dissolved in the number $N_{0}$ of solvent molecules (S). Molecules can be any type, such as high molecular weight linear polymers, star polymers, low molecular weight polyfunctional molecules, etc. Each molecule carries the number *f* of functional groups A (*functionalityf*), which can form interchain cross-links made up of variable number *k* of A-groups (*multiplicityk*) [4,22,23].

We are based on the lattice-theoretical picture of polymer solutions [40], and divide the system volume *V* into cells of size *a* of the solvent molecule, each of which is assumed to accommodate a statistical repeat unit of the reactive molecules. The volume of a reactive molecule is then given by *n*, and that of a solvent molecule is $n_{0}\equiv 1$ in the unit of the cell volume. We assume incompressibility of the solution, so that we have $\Omega =nN+N_{0}$ for the total volume. The volume fraction of each component is then given by ϕ=nN/Ω for the reactive molecule, $\phi_{0}=N_{0}/\Omega$ for the solvent. In terms of the functional groups, the number concentration of A-groups on the reactive molecules is ψ=fϕ/n.

**Figure 1:**
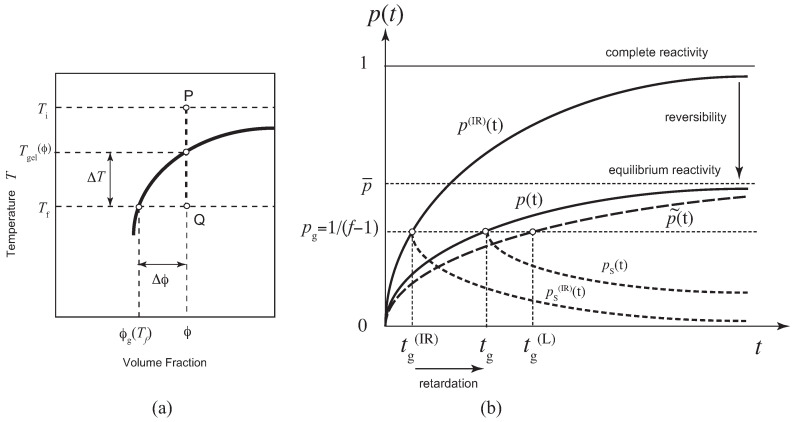
Fundamental picture of the problem studied in this paper. (**a**) Schematic diagram for a temperature (or concentration) jump experiment. A polymer solution is kept in equilibrium at sufficiently high temperature $T_{i}$ in the sol state P. At time t=0, it is quenched to a low temperature $T_{f}\left(\equiv T\right)$ in the gel region Q, and then time development of cross-linking reaction is observed. The quench depth is defined by $\Delta T\equiv T_{\mathrm{gel}}-T$. For a concentration jump experiment, polymers are quickly mixed with solvent at a constant temperature *T* to reach the state Q. The concentration depth is defined by $\Delta \phi \equiv \phi -\phi_{g}$. (**b**) Reactivity of functional groups for pairwise cross-linking schematically shown as a function of the time after the reaction is started: p(t) exact solution, $p^{\left(\mathrm{IR}\right)}\left(t\right)$ its irreversible limit, and $\tilde{p}\left(t\right)$ linear approximation. The gelation time $t_{g}$ is found by the gel-point condition for the reactivity $p\left(t_{g}\right)=1/\left(f-1\right)$. Due to the backward reaction, there is a retardation time $t_{g}-t_{g}^{\left(\mathrm{IR}\right)}$ for reversible cross-linking. The dotted lines show the reactivity of the sol part after the gel point is passed. They are considered in this paper on the basis of Flory’s picture.

The fundamental picture of our research problem is summarized in Figure 1. We study the time development of the solution after its temperature is changed suddenly from the initial one $T_{i}$ to the final one $T\equiv T_{f}$ (temperature jump), or after the molecules are quickly dissolved to the solvent in the preparation of the solution with concentration ϕ at a constant temperature *T* (concentration jump) (see Figure 1a). The temperature quench depth is defined by $\Delta T\equiv T_{\mathrm{gel}}-T$, while the concentration quench depth is $\Delta \phi \equiv \phi -\phi_{g}$, where $T_{\mathrm{gel}}$ and $\phi_{g}$ are their values on the sol–gel transition line. For simplicity, we consider in this paper only cold-setting gels. For heat-setting gels, other factors, such as dehydration, polymer conformation change, temperature activation of the functional groups, etc., must be considered in addition to simple cross-linking. Hence, they lie beyond the scope of the present paper.

After a temperature (or concentration) jump, cross-link reactions leading to network formation proceed. Let $n_{k}\left(t\right)$ be the number of the cross-link junctions of multiplicity *k* at time *t*. Because each junction includes the number *k* of the functional groups A, the probability $p_{k}\left(t\right)$ for an arbitrarily chosen functional group to belong to the junction of multiplicity *k* is related to the number of junctions by the equation

(3) $$\psi p_{k}\left(t\right)/k=n_{k}\left(t\right)$$

After a long time, the solution reaches its equilibrium state with equilibrium reactivity ${\bar{p}}_{k}\equiv p_{k}\left(t\to \infty \right)$. Figure 1b schematically shows the time development of $p\left(t\right)\equiv p_{2}\left(t\right)$, its linearized approximation $\tilde{p}\left(t\right)$, irreversible counterpart $p^{\left(\mathrm{IR}\right)}\left(t\right)$, reactivity of the sol part $p_{s}\left(t\right)$ in the post-gel region. Because of the reverse reaction (dissociation of cross-links), there is a retardation in the gelation time from the irreversible limit.

In our previous work [24], we studied in detail thermoreversible gelation and phase separation in solutions capable of unary (self) cross-linking. We started from the equilibrium condition

(4) $$\frac{\psi {\bar{p}}_{k}/k}{{\left(\psi {\bar{p}}_{1}\right)}^{k}}=K_{k}\left(T\right)$$

where $K_{k}\left(T\right)$ is the equilibrium constant, $p_{1}\left(t\right)$ is the probability for a functional group to remain free from reaction, and ${\bar{p}}_{1}$ is its equilibrium value. This equilibrium condition leads to

(5) $$\psi {\bar{p}}_{k}=kK_{k}{\bar{z}}^{k}$$

for the reactivity in terms of the number of free groups $\bar{z}\equiv \psi {\bar{p}}_{1}$. From the normalization condition of ${\bar{p}}_{k}$, we find the conservation law

(6) $$\psi =\bar{z}u\left(\bar{z}\right)$$

where

(7) $$u\left(\bar{z}\right)=\sum_{k\geq 1}kK_{k}{\bar{z}}^{k-1}$$

### 2.1. Pairwise Cross-Linking

Let us first consider the simplest case of pairwise cross-linking reaction

(8) $$2A_{1}⇆A_{2}$$

for which *k* takes only two values: k=1 (free), and k=2 (bound). Cross-linking by a covalent bond is included as the irreversible limit of such a reaction. For such a simple second order reaction, we can find the exact solution of the rate equation. Therefore, the model provides a good starting point for the study of more complex cross-linking.

Let us write as $p_{2}\equiv p,\;p_{1}=1-p$. Probability *p* is the conventional reactivity of the cross-linking reaction. The time development kinetic equation for the number $n_{k}$ of cross-link junctions can be written as

(9) $$\frac{dn_{2}\left(t\right)}{dt}=\alpha n_{1}{\left(t\right)}^{2}-\beta n_{2}\left(t\right)$$

and hence we have

(10) $$\frac{dp(t)}{dt}=2\psi \alpha {\left(1-p\right)}^{2}-\beta p\left(t\right)$$

where α and β are the rate constant of the forward reaction and backward reaction. (Throughout this paper, we avoid conventional symbols $k_{f}$ and $k_{b}$ to prevent confusion with multiplicity *k*.) By using the scaled time τ≡2ψαt, we have a simple second order equation

(11) $$\frac{dp}{d\tau }=g\left(p\right)$$

where

(12) $$g\left(p\right)\equiv {\left(1-p\right)}^{2}-\frac{p}{2K_{2}\psi }$$

Here, a new constant

(13) $$K_{2}=\alpha /\beta$$

is the equilibrium constant written in terms of the rate constants.

The solution of this equation with the initial condition p(0)=0 is given by

(14) $$p\left(\tau \right)=\frac{\eta^{\left(+\right)}\eta^{\left(-\right)}\left(1-e^{-\gamma \tau }\right)}{\eta^{\left(+\right)}-\eta^{\left(-\right)}e^{-\gamma \tau }}$$

where

(15) $$\eta^{\left(\pm \right)}=\frac{1}{4K_{2}\psi }\left\{1+4K_{2}\psi \pm \sqrt{1+8K_{2}\psi }\right\}$$

and

(16) $$\gamma \equiv \eta^{\left(+\right)}-\eta^{\left(-\right)}=\frac{1}{2K_{2}\psi }\sqrt{1+8K_{2}\psi }$$

The equilibrium reactivity is given by $\bar{p}=\eta^{\left(-\right)}$. Therefore, γ has the meaning of relaxation time of the reversible reaction, i.e., the time necessary for the system to reach its chemical equilibrium. A detailed derivation of (14) is given in Appendix A.

**Figure 2:**
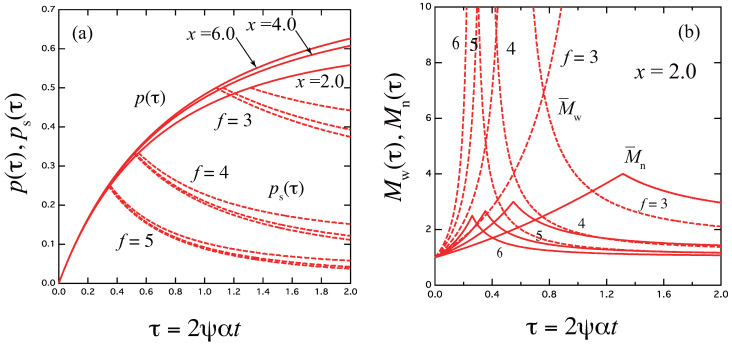
(**a**) Reactivity p(τ) for the functionality f=3,4,5 plotted against the dimensionless scaled time τ≡2ψαt for various scaled concentration $x\equiv K_{2}\left(T\right)\psi =2,4,6$ (solid lines). The relaxation time is independent of *f* as a function of τ, so that p(τ) depends only on *x*. Broken lines are the reactivity of the sol part $p_{s}\left(\tau \right)$ in the post-gel region. (**b**) The weight-average molecular weight (broken lines) and the number-average molecular weight (solid lines) plotted against the scaled time τ for the concentration x=2.0. The number-averages are finite at the gel point.

The result is drawn in Figure 2a for the functionality f=3,4,5. In Figure 2b, the weight- and number-average molecular weight of the three-dimensional cross-linked polymers (${\bar{M}}_{w}$ and ${\bar{M}}_{n}$ in the unit of the molecular weight of the primary molecules) are plotted against time. They are explicitly given by [33]

(17) $${\bar{M}}_{w}\left(\tau \right)=\frac{1+p(\tau )}{1-f^{\prime }p\left(\tau \right)},\;{\bar{M}}_{n}\left(\tau \right)=\frac{1}{1-fp(\tau )/2}$$

In the post-gel region where the gel point is passed, the reactivity of the sol part and that of the gel part become different. We have calculated the former on the basis of Flory’s picture. However, there are other possibilities [35,36,37,38]. In this paper, we focus on the process of approaching the gel point, and prevent discussion on the post-gel regime.

In Figure 2b, the weight-average molecular weight (broken lines) and the number-average molecular weight (solid lines) are plotted against the scaled time τ for the combined variable (scaled concentration) $x\equiv K_{2}\psi =2.0$. The number-average remains finite at the gel point.

Because the relaxation time $t_{R}$ is defined by the speed to reach the equilibrium state, it is given by 1/γ. In terms of the bare time, we have

(18) $$\frac{1}{t_{R}}=2\psi \alpha \gamma =\frac{\alpha }{K_{2}}\sqrt{1+8K_{2}\psi }=\beta \sqrt{1+8K_{2}\psi }$$

In the limit of irreversible reaction where β→0 with finite α, γτ is small, and $\eta^{\left(\pm \right)}\to 1$, so that we go back to the Stockmayer’s result [33,34] (see $p^{\left(\mathrm{IR}\right)}\left(t\right)$ in Figure 1b)

(19) $$p\left(t\right)=p^{\left(\mathrm{IR}\right)}=\frac{2\psi \alpha t}{1+2\psi \alpha t}$$

So far, the reaction has been assumed to occur independently with equal probability for any functional group (*assumption of equal reactivity*). Now, we employ an additional assumption such that all cross-linked three-dimensional molecules take tree forms. Cycle formation is not considered. Then, the conventional tree statistics [33,34,40,41,42,43] give the gel point

(20) $$p_{g}=1/\left(f-1\right)$$

for the reactivity. Substituting this value for p(τ) in (14), we find for the gelation time

(21) $$t_{g}\left(x\right)=t_{R}\left(x\right)Q\left(x\right)$$

where

(22) $$Q\left(x\right)\equiv \ln\left\{\frac{\eta^{\left(-\right)}\left(x\right)\left[f^{\prime }\eta^{\left(+\right)}\left(x\right)-1\right]}{\eta^{\left(+\right)}\left(x\right)\left[f^{\prime }\eta^{\left(-\right)}\left(x\right)-1\right]}\right\}$$

is a function of a combined variable $x\equiv K_{2}\left(T\right)\psi$ of the temperature and concentration. Since this factor Q(x) drastically depends upon the quench depth ΔT, or Δϕ, in the experiments near the sol–gel transition point, we refer to it as *thermodynamic factor*. In particular, the gel-point condition (20) is equivalent to $f^{\prime }\eta^{\left(-\right)}\left(x\right)-1=0$, so that Q(x) goes logarithmically to infinity as *x* approaches the critical value $x_{g}=f^{\prime }/2{f^{\prime \prime }}^{2}$ at the gel point. (In what follows, we use abbreviated notations $f^{\prime }\equiv f-1,f^{\prime \prime }\equiv f-2,\cdots$ for simplicity.) Near the gel point, we have $x\equiv x_{g}\left(1+\epsilon \right)\;\left(\epsilon <<1\right)$. A lengthy calculation leads to the form

(23) $$Q\left(x\right)≃\ln\left\{{\left(\frac{ff^{\prime \prime }}{{f^{\prime }}^{2}}\right)}^{2}\frac{1}{\epsilon }\right\}≃-\ln\epsilon +\mathrm{finite}\;\mathrm{const}.$$

Therefore, in the limit of shallow quenching, we have to wait for a logarithmically long time for the solution to turn into a gel.

As for the relaxation time, we have to notice that $t_{R}$ can be expressed in terms of the equilibrium reactivity. The solution of the conservation law (6) for a pairwise cross-linking is given by

(24) $$\bar{z}=\frac{1}{4K_{2}}\left\{-1+\sqrt{1+8K_{2}\psi }\right\}$$

We therefore have ${\bar{p}}_{2}/{\bar{p}}_{1}=2K_{2}\bar{z}$, and hence

(25) $$\frac{1}{t_{R}}=\beta \left(1+2\frac{{\bar{p}}_{2}}{{\bar{p}}_{1}}\right)$$

We will have some extensions of this formula in the following sections for the study of multiple cross-links.

We next consider retardation of the gelation time due to the reversible reaction. We start from the gelation time (21) in the form

(26) $$\psi \alpha t_{g}=\frac{x}{\sqrt{1+8x}}Q\left(x\right)$$

and take β→0 limit while α is kept finite. Because *x* takes a large value, we expand the right hand side of this equation in powers of 1/x. After a quite a lengthy calculation, we find

(27) $$t_{g}=\frac{1}{2\alpha f^{\prime \prime }\psi }\left(1+\frac{R_{f,2}}{x}+O\left(\frac{1}{x^{2}}\right)\right)$$

where the front factor

(28) $$t_{g}^{(\mathrm{IR})}\equiv \frac{1}{2\alpha f^{\prime \prime }\psi }$$

is the gelation time of an irreversible reaction (covalent bonding). It can directly be obtained from Stockmayer’s form (19) by fixing $p\left(t\right)=1/f^{\prime }$. The first correction due to reversible reaction is O(1/x). Its coefficient is found to be

(29) $$R_{f,2}=\frac{3f-4}{6{f^{\prime \prime }}^{2}}$$

Let us refer to it as *retardation coefficient*. In the next section, we shall derive the retardation coefficient $R_{f,k}$ for the cross-linking with arbitrary multiplicity *k*.

**Figure 3:**
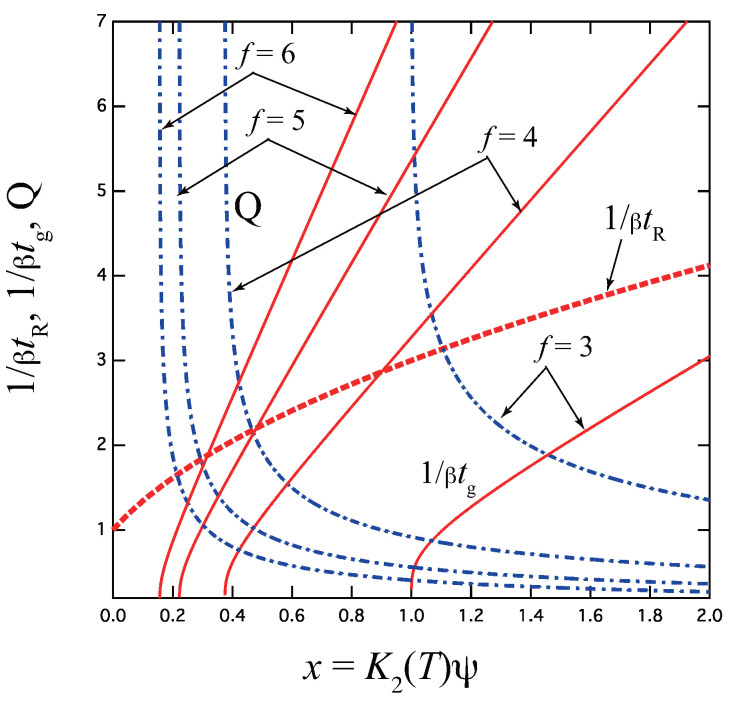
The reciprocal gelation time $1/\beta t_{g}$ (solid lines), reciprocal relaxation time $1/\beta t_{R}$ (broken lines), and thermodynamic factor *Q* (broken dotted lines) plotted against a combined variable $x\equiv K_{2}\left(T\right)\psi$ for various functionality *f*. $1/\beta t_{g}$ goes logarithmically to zero near the equilibrium sol–gel transition point $x_{g}=f^{\prime }/2{f^{\prime \prime }}^{2}$, while it is proportional to *x* at high concentration regions. $t_{R}$ is independent of *f*, and remains finite at the gel point, while *Q* goes to infinity.

To compare with experimental data, we plot in Figure 3 the reciprocal of the gelation time

(30) $$\frac{1}{\beta t_{g}}=\frac{\sqrt{1+8x}}{Q(x)}\equiv \Gamma \left(x\right)$$

as a function of the combined variable *x* for varied functionality *f*.

Near the sol–gel transition point $x=x_{g}$, it goes logarithmically to zero as

(31) $$\Gamma \left(x\right)≃\frac{\sqrt{1+8x_{g}}}{\ln\left(1/\Delta x\right)}$$

For larger values of the functionality *f*, all plots remain qualitatively the same although quantitatively very different. They approach much closer to the vertical axis.

Experimental data [16,17] on PVA in a mixed solvent of water and DMSO suggest that the gelation time near the transition point obeys a power low of concentration with the power index 2. The paper attributed this observation to the binary collision of the polymers at the overlap concentration, which is necessary for the formation of microcrystalline junctions for PVA solutions. In the present models of cross-linking reaction among functional groups, the gel point is located at a much lower concentration than the overlap concentration.

For sufficiently high *x*, it approaches the irreversible limit

(32) $$\Gamma \left(x\right)≃2f^{\prime \prime }x$$

In this limit, the gelation time is separable into a product of the temperature factor λ(T) and the concentration factor ψ=fϕ/n. The linear behavior in the high concentration region has not been experimentally reported.

So far, the gelation time has been derived from the exact solution of the rate equation. The only assumption is that the gel point is assumed to be given by the conventional tree approximation. We now examine the validity of the linearized equation of (11) before moving to more complex cross-links with variable multiplicity. As time goes on, the polymer solution approaches its equilibrium state. Assuming that it is sufficiently close to equilibrium, let the reactivity be

(33) $$p\left(t\right)=\bar{p}\left(1+\xi \left(t\right)\right)$$

and consider only linear terms of the deviation ξ(t) in (11). We have

(34) $$\frac{d\xi }{d\tau }=g^{\prime }\left(\bar{p}\right)\xi$$

where

(35) $$g^{\prime }\left(\bar{p}\right)=-\left\{2\left(1-\bar{p}\right)+\frac{1}{2K_{2}\psi }\right\}$$

is the derivative of $g(\bar{p})$. Substituting the equilibrium value of *p* leads to

(36) $$g^{\prime }\left(\bar{p}\right)=-\frac{1}{2K_{2}\psi }\left(1+2\frac{{\bar{p}}_{2}}{{\bar{p}}_{1}}\right)$$

Hence, the relaxation time in the linear approximation agrees with the exact one. If we assume p(0)=0, the reactivity in the linear approximation takes the form (see Figure 1b)

(37) $$\tilde{p}\left(t\right)=\bar{p}\left(1-e^{-t/t_{R}}\right)$$

The gel-point condition $p\left(t_{g}\right)=1/f^{\prime }$ then leads to (21), but with Q(x) of slightly different form

(38) $$Q\left(x\right)=\ln\left\{\frac{f^{\prime }\eta^{\left(+\right)}\left(x\right)}{f^{\prime }\eta^{\left(-\right)}\left(x\right)-1}\right\}≃-\ln\epsilon +\mathrm{finite}\;\mathrm{const}.$$

for the thermodynamic factor leading to the same logarithmic divergence. Unfortunately, the linear approximation does not give a correct irreversible limit, so that retardation effect is impossible to study.

### 2.2. Fixed-Multiplicity Model

**Figure 4:**
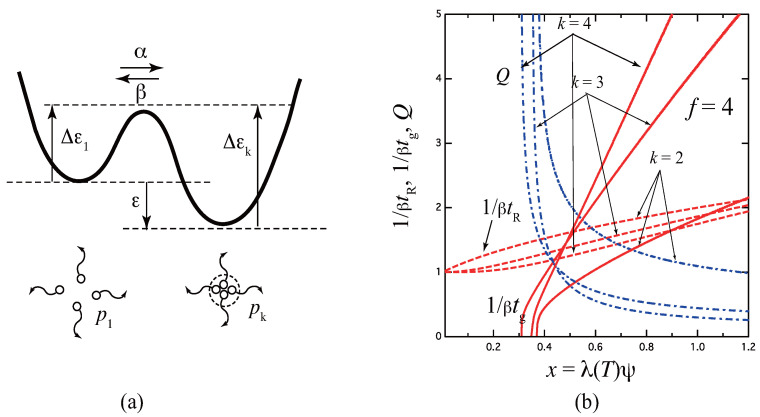
(**a**) Schematic picture of the fixed-multiplicity cross-linking model with multiplicity *k*. (**b**) The reciprocal gelation time $1/\beta t_{g}$, the reciprocal relaxation time $1/\beta t_{R}$, and the thermodynamic factor Q(x) plotted against a combined variable x≡λ(T)ψ for various multiplicity *k* for the tetra-functional molecules (f=4). $1/\beta t_{g}$ goes logarithmically to zero near the equilibrium sol–gel transition point given by $x_{g}=f^{\prime }k^{\prime }/k^{1/k^{\prime }}{\left(f^{\prime }k^{\prime }-1\right)}^{k/k^{\prime }}$, while it approaches the irreversible limit $∼x^{k^{\prime }}$ at a high concentration region. Note that the gel point concentration changes non-monotonically as a function of the multiplicity.

Because most of physical gels have multiple cross-link junctions, let us next consider the effect of cross-link multiplicity. We first study an extreme case where simultaneous formation of *k* junctions takes place

(39) $$kA_{1}⇄A_{k}$$

before we move onto a more complex case of stepwise association. A functional group is either free (k=1) or reacted (k≥2). Let us refer to it as a *fixed-multiplicity model* (see Figure 4).

We have a rate equation for the fixed-multiplicity reversible reaction

(40) $$\frac{dn_{k}\left(t\right)}{dt}=\alpha n_{1}{\left(t\right)}^{k}-\beta n_{k}\left(t\right)$$

Or, equivalently

(41) $$\frac{dp_{k}\left(t\right)}{dt}=\alpha k\psi^{k^{\prime }}p_{1}{\left(t\right)}^{k}-\beta p_{k}\left(t\right)$$

($k^{\prime }\equiv k-1$). Hence, we have

(42) $$\frac{dp}{d\tau }=g\left(p\right)$$

where $p\equiv p_{k},p_{1}=1-p$. Time is scaled as

(43) $$\tau \equiv \alpha k\psi^{k^{\prime }}t$$

and

(44) $$g\left(p\right)\equiv {\left(1-p\right)}^{k}-\beta^{\prime }p$$

The coefficient β of the reverse reaction is changed to

(45) $$\beta^{\prime }\equiv \frac{\beta }{\alpha k\psi^{k^{\prime }}}=\frac{1}{kK_{k}\psi^{k^{\prime }}}$$

where

(46) $$K_{k}=\frac{\alpha }{\beta }$$

is the equilibrium constant.

In the study of heat-setting β-Lactoglobulin, Tobitani and Ross-Murphy [20,21] solved this equation in the irreversible limit of β→0. The rate equation can easily be integrated into this limit. We find

(47) $$\tau =\frac{1}{k^{\prime }}\left\{\frac{1}{{\left(1-p\right)}^{k^{\prime }}}-\frac{1}{{\left(1-p\left(0\right)\right)}^{k^{\prime }}}\right\}$$

The relaxation is not exponential, but obeys a power law. Let us assume that all functional groups are free in the initial state (p(0)=0).

In our previous study of gelation with cross-links of variable multiplicity [23,24], we showed that the gel point is in general given by the condition

(48) $$1-f^{\prime }\left({\langle k\rangle }_{w}-1\right)=0$$

where

(49) $${\langle k\rangle }_{w}\equiv \sum_{k\geq 1}kp_{k}$$

is the average multiplicity of the junctions. The average $\sum_{k}\left(k-1\right)p_{k}={\langle k\rangle }_{w}-1$ was referred to as a *branching number* [23,24]. In the case of the fixed-multiplicity model, the gel point is found by the condition

(50) $$p_{k}\left(t_{g}\right)=\frac{1}{f^{\prime }k^{\prime }}\equiv p_{g}$$

Substituting this value into (47), we find

(51) $$\frac{1}{t_{g}^{\left(\mathrm{IR}\right)}}=\alpha k\psi^{k^{\prime }}\frac{k^{\prime }{\left(1-p_{g}\right)}^{k^{\prime }}}{1-{\left(1-p_{g}\right)}^{k^{\prime }}}\equiv A\left(T\right)\phi^{k^{\prime }}$$

Therefore, it turns out that temperature and concentration are separable for irreversible gelation. The temperature factor is

(52) $$A\left(T\right)=\alpha \left(T\right){\left(\frac{f}{n}\right)}^{k^{\prime }}\frac{kk^{\prime }}{{\left[f^{\prime }k^{\prime }/\left(f^{\prime }k^{\prime }-1\right)\right]}^{k^{\prime }}-1}$$

It goes back to (28) for the irreversible pairwise cross-linking of k=2.

Let us study reversible rate equation. Because rigorous integration of (42) is difficult, let us employ the linear approximation. The linearized kinetic equation leads to the solution

(53) $$\tilde{p}\left(\tau \right)=\bar{p}\left(1-e^{-\gamma \tau }\right)$$

where

(54) $$\gamma \equiv -g^{\prime }\left(\bar{p}\right)=k{\left(1-\bar{p}\right)}^{k^{\prime }}+\beta^{\prime }$$

and hence the relaxation time is

(55) $$\frac{1}{t_{R}}=\alpha k\psi^{k^{\prime }}\gamma =\beta \left(1+k^{2}K_{k}{\bar{z}}^{k^{\prime }}\right)=\beta \left(1+k\frac{{\bar{p}}_{k}\left(\bar{z}\right)}{{\bar{p}}_{1}\left(\bar{z}\right)}\right)$$

by using equilibrium values of the reactivities. They can be written as functions of the solution $\bar{z}$ of the conservation Equation (6) with

(56) $$u\left(\bar{z}\right)\equiv 1+kK_{k}{\bar{z}}^{k^{\prime }}$$

The relaxation time similar form to (55) was first proposed by Kresheck et al. [44] (referred to as KHDS), and later by Muller [45] for the self-assembled micelle formation in solutions of surfactant molecules studied by temperature-jump experiments. The KHDS form was derived under the assumption that, in the stepwise association of the surfactant molecules, the last step is slowest compared to the intermediate steps, and hence it is the rate-controlled step.

Substituting the gel-point condition (48) into the linear solution (53), we find the gelation time takes the same form as (21), but with the different thermodynamic factor

(57) $$Q\left(\bar{z}\right)=\ln\left(\frac{f^{\prime }k^{\prime }{\bar{p}}_{k}\left(\bar{z}\right)}{f^{\prime }k^{\prime }{\bar{p}}_{k}\left(\bar{z}\right)-1}\right)$$

The equilibrium gel point is given by the condition $1-f^{\prime }k^{\prime }{\bar{p}}_{k}\left(\bar{z}\right)=0$ and hence

(58) $${\left(K_{k}\left(T\right)\psi^{k^{\prime }}\right)}_{g}=\frac{{\left(f^{\prime }k^{\prime }\right)}^{k^{\prime }}}{k{\left(f^{\prime }k^{\prime }-1\right)}^{k}}$$

We find again a logarithmic divergence of the thermodynamic factor near the sol–gel transition line.

To find specific results, let us assume that the equilibrium constant takes a form [23]

(59) $$K_{k}\left(T\right)=\lambda {\left(T\right)}^{k^{\prime }}$$

where λ(T) is a binding constant per one functional group. The conservation law becomes

(60) $$x\equiv \lambda \psi =\bar{z}\left(1+k{\bar{z}}^{k^{\prime }}\right)$$

where $\bar{z}\equiv x\left(1-{\bar{p}}_{k}\right)$ is the scaled concentration of the free groups. Solving this equation for $\bar{z}$, and substituting the solution to

(61) $$\tau_{g}\equiv \alpha k\psi^{k^{\prime }}t_{g}=\frac{kx^{k^{\prime }}Q\left(\bar{z}\right)}{1+k{\bar{p}}_{k}\left(\bar{z}\right)/{\bar{p}}_{1}\left(\bar{z}\right)}$$

we find the gelation time as functions of *x*. Let us plot it in our standard form

(62) $$\Gamma \left(x\right)\equiv \frac{1}{\beta t_{g}}=\left(1+k^{2}{\bar{z}}^{k^{\prime }}\right)\ln\left(\frac{f^{\prime }k^{\prime }k{\bar{z}}^{k}}{f^{\prime }k^{\prime }k{\bar{z}}_{x}^{k}-1}\right)$$

where $\bar{z}=\bar{z}\left(x\right)$ is the solution of the conservation law (60).

Figure 4b plots $1/\beta t_{g}$, $1/\beta t_{R}$, and *Q* as functions of *x* for varied multiplicity *k* for tetra-functional molecules f=4 as a typical example. Multiplicity k=2 goes back to the pairwise association studied in the previous section. All curves diverge logarithmically as *x* approaches the critical value $x_{g}=f^{\prime }k^{\prime }/k^{1/k^{\prime }}{\left(f^{\prime }k^{\prime }-1\right)}^{k/k^{\prime }}$ from above. For f=4, we know that the gel point is not a monotonically decreasing function of *k*, but takes a minimum at k=3 (see Figure 7.5 (a) in the reference [4]).

The retardation coefficient $R_{f,k}$ for *f*-functional primary molecules with cross-link multiplicity *k* can be found in a similar method of expanding $t_{g}$ in powers of the reverse rate constant β. We find

(63) $$t_{g}=t_{g}^{\left(\mathrm{IR}\right)}\left\{1+\frac{R_{f,k}}{x^{k^{\prime }}}+O\left(\frac{1}{x^{2k^{\prime }}}\right)\right\}$$

with

(64) $$R_{f,k}=\frac{{\left(1-p_{g}\right)}^{2k-1}+\left(2k-1\right)p_{g}-1}{2k\left(2k-1\right)\left[1-{\left(1-p_{g}\right)}^{k^{\prime }}\right]{\left(1-p_{g}\right)}^{k}}$$

where $t_{g}^{\left(\mathrm{IR}\right)}$ is the irreversible gelation time (51) by Tobitani–Ross–Murphy [20]. Detailed calculation is given in Appendix B.

### 2.3. Stepwise Association

In most physical gels, we expect that cross-linking proceeds via step-by-step association of the free functional groups:

(65) $$A_{1}+A_{k-1}⇆A_{k}\;\left(k=2,3,\cdots s\right)$$

**Figure 5:**
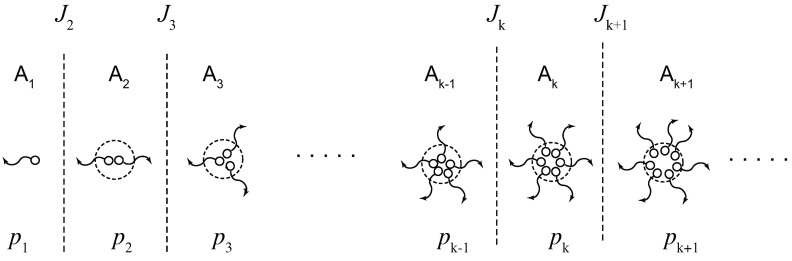
Schematic picture to construct the rate equation for a stepwise association during cross-linking. The reactivity $p_{k}$ is the probability for an arbitrarily chosen functional group to belong to a cross-link junction of the multiplicity *k*. The flux $J_{k}$ is the number concentration of the junctions growing from k−1 to *k* in a unit time.

In some physical gels, a particular value of the multiplicity *k* is most stable, while in others cross-links are monotonically destabilized with increasing *k*. The gelation time and physical properties of the networks thus depend on the stepwise association constants. Let us therefore study thermoreversible gelation by such step-by-step cross-linking for a given set of stepwise constants as a model reversible gelation (see Figure 5).

The rate equation is described by

(66a)dnk(t)dt=Jk−Jk+1(66b)=−{βk+αkn1(t)}nk(t)+βk+1nk+1(t)+αk−1n1(t)nk−1(t)

for k≥2, and

(67a)dn1(t)dt=−2J2−∑k=3sJk(67b)=−2α1n1(t)2+2β2n2(t)+∑k=3s{βknk(t)−αk−1n1(t)nk−1(t)}

for k=1. Here,

(68) $$J_{k}\left(t\right)\equiv \alpha_{k-1}n_{1}\left(t\right)n_{k-1}\left(t\right)-\beta_{k}n_{k}\left(t\right)$$

for k≥2 is the flux between k−1 and *k* state ($J_{1}\equiv 0$), i.e., the number of k−1 junctions changing to *k* junctions per unit time as a result of forward and backward reaction with the rate constant $\alpha_{k}$ and $\beta_{k}$ (see Figure 5).

Similar rate equations were proposed by Aniansson and Wall [46,47,48] for the study of micelle formation in surfactant solutions. In our gelation problem, associating groups are attached to the polymer main chains (indicated by arrows), while in the micellization problem they move freely. However, within the assumption of equal reactivity, the basic kinetics governing the association process can be regarded as fundamentally the same in both cases.

Let us first transform these equations by using reactivity $p_{k}\left(t\right)$ in order to find the equilibrium solution easily. Using Equation (3), we find

(69) $$\frac{dp_{k}\left(t\right)}{dt}=-\left\{\beta_{k}+\alpha_{k}\psi p_{1}\left(t\right)\right\}p_{k}\left(t\right)+\frac{k}{k+1}\beta_{k+1}p_{k+1}\left(t\right)+\frac{k}{k-1}\alpha_{k-1}\psi p_{1}\left(t\right)p_{k-1}\left(t\right)$$

for k≥2, and

(70) $$\frac{dp_{1}\left(t\right)}{dt}=-2\alpha_{1}\psi p_{1}{\left(t\right)}^{2}+\beta_{2}p_{2}\left(t\right)+\sum_{k=3}^{s}\left\{\frac{\beta_{k}}{k}p_{k}\left(t\right)-\frac{\alpha_{k-1}}{k-1}\psi p_{1}\left(t\right)p_{k-1}\left(t\right)\right\}$$

for k=1.

Let us next confirm that the equilibrium distribution (5) satisfies these kinetic equations. On substitution of (5) into (69), we find that the relation

(71) $$\left(\beta_{k}K_{k}-\alpha_{k-1}K_{k-1}\right)-\left(\alpha_{k}K_{k}-\beta_{k+1}K_{k+1}\right)\bar{z}=0$$

must be fulfilled. We therefore propose the *detailed balance condition* such that the equlibrium constants satisfy the relation

(72) $$K_{k}=\frac{\alpha_{k-1}}{\beta_{k}}K_{k-1}$$

By repeated use of this relation, we have a well-known relation

(73) $$K_{k}=\frac{\alpha_{k-1}\alpha_{k-2}}{\beta_{k}\beta_{k-1}}K_{k-2}=\cdots =\lambda_{k}\lambda_{k-1}\cdots \lambda_{2}$$

between the equilibrium constants $K_{k}$ and the stepwise association constants

(74) $$\lambda_{k}\equiv \frac{\alpha_{k-1}}{\beta_{k}}$$

for k−1 and *k* state. The kinetic Equations (69) and (70), together with the detailed balance conditions (73), provide a complete set to find the solution for the gelation time. The gel-point condition is given by (48).

## 3. Results

### 3.1. Three-State Model

First, we study the three-state model of cross-linking. In this model, we have free (k=1), double (k=2), and triple (k=3) cross-link junctions. Some biopolymers form cross-links of either double helix or triple helix [2,49,50]. Depending on the environmental condition, there is a competition between them. We can study such competition in forming cross-links by using a three-state rate equation.

The kinetic equations are

(75a)dp1dt=−2α1p12+(β2−12α2ψp1)p2+β33p3(75b)dp2dt=2α1ψp12−(β2+α2ψp1)p2+2β33p3(75c)dp3dt=3α22ψp1p2−β3p3

The conservation law of the total number of functional groups is described by the normalization condition $p_{1}+p_{2}+p_{3}=1$. The equilibrium solutions are given by $\psi {\bar{p}}_{1}=\bar{z},\psi {\bar{p}}_{2}=2K_{2}{\bar{z}}^{2},\psi {\bar{p}}_{3}=3K_{3}{\bar{z}}^{3}$. Detailed balance conditions lead to the relation $K_{2}=\alpha_{1}/\beta_{2}\equiv \lambda_{2}$, and $K_{3}=\alpha_{1}\alpha_{2}/\beta_{2}\beta_{3}\equiv \lambda_{2}\lambda_{3}$.

Let us first eliminate triple association $p_{3}$ by using the normalization condition. Then, we transform the kinetic equation in terms of the deviation $\xi_{1},\xi_{2}$ from the equilibrium state defined by $p_{k}\left(t\right)\equiv {\bar{p}}_{k}\left(1+\xi_{k}\left(t\right)\right)$. After a lengthy calculation, we find

(76) $$\frac{d}{dt}\left[\begin{array}{c} \xi_{1}\\ \xi_{2} \end{array}\right]=-\left[\begin{array}{cc} a_{1,1} & a_{1,2}\\ a_{2,1} & a_{2,2} \end{array}\right]\left[\begin{array}{c} \xi_{1}\\ \xi_{2} \end{array}\right]+\left[\begin{array}{c} f_{1}\left(\xi_{1},\xi_{2}\right)\\ f_{2}\left(\xi_{1},\xi_{2}\right) \end{array}\right]$$

where

(77a)a1,1≡β33+4α1z¯+α2K2z¯2(77b)a1,2≡K2z¯2β33−2β2+α2z¯(77c)a2,1≡1K2z¯β33−2α1z¯+α2K2z¯2(77d)a2,2≡β2+β323+λ3z¯

for the linear terms, and

(78a)f1(ξ1,ξ2)≡−(2α1ξ1+α2K2z¯ξ2)z¯ξ1(78b)f2(ξ1,ξ2)≡(α1ξ1−α2K2z¯ξ2)ξ1/K2

for the nonlinear (quadratic) terms.

We can numerically solve these equations for a given set of rate constants. However, here we confine to the linear approximation in order to obtain physical picture on the factors controlling the gelation time. Nonlinear terms are used only for the stability analysis of the linear solution.

Let us find the eigen-values γ of the matrix $\hat{A}\equiv \left(a_{i,j}\right)$. The equation to find them is

(79) $$\gamma^{2}-A\left(\bar{z}\right)\gamma +B\left(\bar{z}\right)=0$$

where

(80a)A(z¯)≡Tr(A^)=β2+β3+(4α1+α2)z¯+α2K2z¯2(80b)B(z¯)≡Det(A^)=β2β3+4α1β3z¯+9α1α2z¯2

Note that $B(\bar{z})$ can be written as

(81) $$B\left(\bar{z}\right)=\beta_{2}\beta_{3}{\left(\bar{z}u\left(\bar{z}\right)\right)}^{\prime }=\beta_{2}\beta_{3}\left(1+2\frac{{\bar{p}}_{2}}{{\bar{p}}_{1}}+3\frac{{\bar{p}}_{3}}{{\bar{p}}_{1}}\right)$$

so that it is regarded as an extension of the KHDS decay rate (55) to the three-state model. (Prime ${}^{\prime }$ indicates differentiation.) The linear relaxation has two modes

(82) $$\gamma^{\left(\pm \right)}\left(\bar{z}\right)\equiv \frac{A(\bar{z})}{2}\left\{1\pm \sqrt{1-\frac{4B(\bar{z})}{A{\left(\bar{z}\right)}^{2}}}\right\}$$

The gel point is given by the condition

(83) $$1-f^{\prime }\left\{p_{2}\left(t\right)+2p_{3}\left(t\right)\right\}=0$$

Using the deviation $\xi_{k}\left(t\right)$, this condition is transformed to

(84) $$\xi_{1}\left(t\right)+K_{2}\bar{z}\xi_{2}\left(t\right)=K_{2}+3K_{3}\bar{z}-1/2f^{\prime }\bar{z}$$

#### 3.1.1. Rate-Determining Step

We first look into the cases where the two rate constants of the backward reaction are largely different. If $\beta_{2}<<\beta_{3}$, the cross-linking is controlled by the first step association (pair formation), and vice versa. In both cases, we have the situation $B/A^{2}<<1$, so that the relaxation time is approximately given by

(85) $$\frac{1}{t_{R}}≃\frac{\beta_{2}\beta_{3}{\left(\bar{z}u\left(\bar{z}\right)\right)}^{\prime }}{\beta_{2}b_{2}\left(\bar{z}\right)+\beta_{3}b_{3}\left(\bar{z}\right)}=\bar{\beta }\left(\bar{z}\right)\left(1+2\frac{{\bar{p}}_{2}}{{\bar{p}}_{1}}+3\frac{{\bar{p}}_{3}}{{\bar{p}}_{1}}\right)$$

where $\bar{\beta }$ is the average of β defined by

(86) $$\frac{1}{\bar{\beta }\left(\bar{z}\right)}\equiv \frac{b_{2}\left(\bar{z}\right)}{\beta_{2}}+\frac{b_{3}\left(\bar{z}\right)}{\beta_{3}}$$

The relaxation time apparently takes an extended KHDS form. Here, we have eliminated $\alpha_{k}$ in favor of $\beta_{k}$ and $\lambda_{k}$ from $A(\bar{z})$, and write

(87) $$A\left(\bar{z}\right)=\beta_{2}b_{2}\left(\bar{z}\right)+\beta_{3}b_{3}\left(\bar{z}\right)$$

by using

(88a)b2(z¯)≡1+4λ2z¯(88b)b3(z¯)≡1+λ3z¯+λ2λ3z¯

If the first association (pairing) is the rate-determining step, the relaxation time is approximately

(89) $$\frac{1}{t_{R}}≃\frac{\beta_{2}}{b_{3}\left(\bar{z}\right)}{\left(\bar{z}u\left(\bar{z}\right)\right)}^{\prime }$$

and goes back to the pairwise association in the limit of $\lambda_{3}\to 0$. If the second associaition (triple junction) is the rate-determining step, the relaxation time is approximately given by

(90) $$\frac{1}{t_{R}}≃\frac{\beta_{3}}{b_{2}\left(\bar{z}\right)}{\left(\bar{z}u\left(\bar{z}\right)\right)}^{\prime }$$

From these results, we may expect the extended KHDS form for the multiplicity more than three

(91) $$\frac{1}{t_{R}}≃\beta \left(1+\frac{1}{{\bar{p}}_{1}}\sum_{k\geq 2}k{\bar{p}}_{k}\right)=\beta \frac{{\langle k\rangle }_{w}}{{\bar{p}}_{1}}$$

with some average β, because the average multiplicity is related to the average branching number of the cross-link junctions.

#### 3.1.2. Quasi-Stationary State Approximation

Let us next consider the special case where the first step reaction is so fast that a stationary state is easily established for the binary cross-linking. Since we have $d\xi_{2}/dt≃0$, the kinetic equation reduces to

(92) $$\frac{d\xi_{1}}{dt}≃-\frac{B(\bar{z})}{a_{2,2}}\xi_{1}$$

The rexation time is then given by

(93) $$\gamma \left(\bar{z}\right)=\frac{\beta_{2}\beta_{3}}{a_{2,2}}{\left(\bar{z}u\left(\bar{z}\right)\right)}^{\prime }$$

The front factor can be regarded as an average backward rate constant $\bar{\beta }$ defined by

(94) $$\frac{1}{\bar{\beta }\left(\bar{z}\right)}\equiv \frac{1}{\beta_{3}}+\frac{2/3+\lambda_{3}\bar{z}}{\beta_{2}}$$

Hence, we find again the same form

(95) $$\frac{1}{t_{R}}=\bar{\beta }\left(\bar{z}\right)\left(1+2\frac{{\bar{p}}_{2}}{{\bar{p}}_{1}}+3\frac{{\bar{p}}_{3}}{{\bar{p}}_{1}}\right)$$

for the relaxation time, with slightly different average of β.

#### 3.1.3. Slow-Mode Approximation

The general solution for the linear equation is given by

(96) $$\left[\begin{array}{c} \xi_{1}\\ \xi_{2} \end{array}\right]=C_{1}e^{1}e^{-\gamma^{\left(-\right)}t}+C_{2}e^{2}e^{-\gamma^{\left(+\right)}t}$$

where $e^{1}$ is the normalized eigen-vector for the eigen-value $\gamma^{\left(-\right)}$ (slow mode) and $e^{2}$ for the fast mode. The constants $C_{1},C_{2}$ can be fixed by the initial conditions $p_{1}\left(0\right)=1,\;p_{2}\left(0\right)=0$. In the slow mode approximation, we take only the slow mode into consideration, and neglect the contribution from the fast mode. Then, the relaxation time is given by $1/t_{R}=\gamma^{\left(-\right)}$, and the gelation time is $t_{g}=t_{R}Q\left(\bar{z}\right)$ with a thermodynamic factor

(97) $$Q\left(\bar{z}\right)=\ln\left\{\frac{2f^{\prime }\bar{z}D_{1}\left(\bar{z}\right)}{f^{\prime }\left({\langle k\rangle }_{w}-1\right)-1}\right\}$$

from (84), where $D_{1}\left(\bar{z}\right)\equiv C_{1}\left(e_{1}^{1}+K_{2}\bar{z}e_{2}^{1}\right)$ is a function of $\bar{z}$, which is non-singular across the gel point. Therefore, we have again logarithmic divergence of the gel time.

#### 3.1.4. Some Numerical Results of the Relaxation Time

In order to find specific results, let us assume that the association constant of the second step is μ times larger than the first one:

(98) $$\lambda_{2}\equiv \lambda ,\;\;\lambda_{3}=\mu \lambda$$

Then, the forward rate constants are given by

(99) $$\alpha_{1}=\beta_{2}\lambda ,\;\;\alpha_{2}=\mu \lambda \beta_{3}$$

The conservation law takes the form

(100) $$x\equiv \lambda \psi =\bar{z}\left(1+\bar{z}+\mu {\bar{z}}^{2}\right)$$

where $\lambda \bar{z}$ is written as $\bar{z}$ for simplicity. Now we have

(101a)A(z¯)=β2(1+4z¯)+β3(1+μz¯+μz¯2)(101b)B(z¯)=β2β3(1+4z¯+9μz¯2)

In particular, for a special case of $\beta_{2}=\beta_{3}\equiv \beta$ the relaxation time is given by

(102) $$\frac{1}{\beta t_{R}}=\frac{1}{2}A\left(\bar{z}\right)\left\{1-\sqrt{1-\frac{4B(\bar{z})}{A{\left(\bar{z}\right)}^{2}}}\right\}$$

with

(103a)A(z¯)=2+(μ+4)z¯+μz¯2(103b)B(z¯)=1+4z¯+9μz¯2

**Figure 6:**
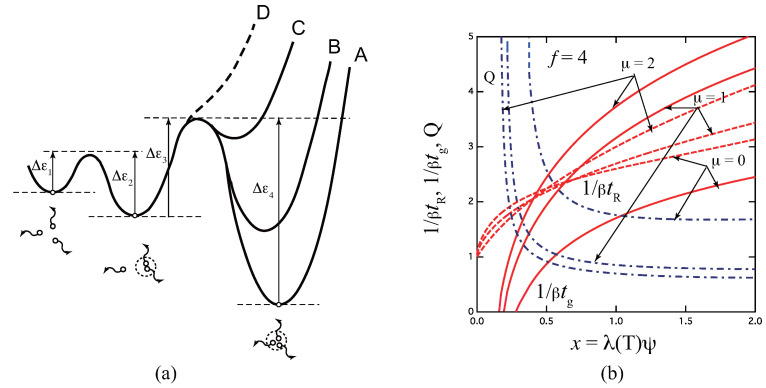
(**a**) Schematic picture of the three-state cross-linking model with multiplicity k=1,2,3. (**b**) The reciprocal gelation time $1/\beta t_{g}$ (solid lines), the reciprocal relaxation time $1/\beta t_{R}$ (broken lines), and the thermodynamic factor Q(x) (broken dotted lines) plotted against a scaled concentration variable x≡λ(T)ψ for various ratio μ=0,1,2 of the stepwise constants for the tetra-functional molecules (f=4). The reciprocal gelation time $1/\beta t_{g}$ goes logarithmically to zero near the equilibrium sol–gel transition point, while it is eventually proportional to $x^{2/3}$ at a high concentration region. With a decrease of μ, the stability of the triple cross-link junction is weakened as shown from A to D in Figure (**a**). For μ=0, the model reduces to the pairwise cross-linking.

Figure 6a shows a schematic free energy surface of association drawn against the reaction coordinates for the three-state model. With a decrease in the ratio μ, the surface curve of the triple junction changes from A to D. For the limit of μ=0 (curve D), the model reduces to the pairwise one. Figure 6b plots the reciprocal gelation time $1/\beta t_{g}$, the reciprocal relaxation time $1/\beta t_{R}$, and the thermodynamic factor Q(x) plotted against a scaled concentration x≡λ(T)ψ for three ratios μ=0,1,2 of the stepwise constants for the tetra-functional molecules (f=4). Due to the existence of k=2 state, relaxation is slower than the fixed-multiplicity model of k=3.

### 3.2. Micellar Cross-Linking

Let us next consider cross-link junctions whose multiplicity is stabilized around a large number $k_{0}$ due to the physical reason of associative force, such as geometrical packing, saturation of the interaction force, etc. Typical examples of thermoreversible gels cross-linked by such micellar junctions are hydrophobically-modified water-soluble polymers (*associating polymers*) [4], for instance, aqueous solutions of polymers with short hydrophobic chains attached at both chain ends (telechelic polymers), such as hydrophobic ethoxylated urethane (called HEUR) [51,52,53], and hydrophobic poly(*N*-isopropylacrylamide) [54,55,56,57]. Hydrophobes form spherical micelles and serve as the network junctions. Triblock coploymers of the type ABA, ABC, etc., for instance, poly(propylene oxide)-poly(ethylene oxide)-poly(propylene oxide) triblock copolymers [58,59,60], are another important example whose cross-links are micelles of various shapes; spherical, cylindrical, planar, etc.; consisting of the block segments.

The size distribution of the cross-links changes as the reaction proceeds step by step, from the unimer (free functional group) dominant one to the final micelle (large junction) dominant one. Such time-development is similar to that observed for the micelle formation in surfactant solutions [44,45,46,47]. The functional groups treated here are, however, attached to the polymer main chains. Therefore, their association takes place under the constraint of chain movement. However, here we assume the equal reactivity, and start from the stepwise kinetic Equations (69) and (70). Their linearized forms are

(104a)dξkdt=−(βk+αkz¯)+βkξk−1+αkz¯ξk+1+(βk−αkz¯)ξ1(k≥2)(104b)dξ1dt=−2α1z¯(2ξ1−ξ2)−∑k≥3βkKkz¯k(ξk−1−ξk+ξ1)

The gel-point condition (48) turns into

(105) $$f^{\prime }\sum_{k\geq 2}\left(k-1\right){\bar{p}}_{k}\xi_{k}\left(t_{g}\right)=1-f^{\prime }\sum_{k\geq 2}\left(k-1\right){\bar{p}}_{k}$$

Following the idea by Aniansson and Wall [46] (referred to as AW), we take an analogy between micelle formation and a heat transfer from one metal to another through a connected thin metal wire. Let us judiciously choose a multiplicity $k_{1}$, below which the population distribution is dominant in the initial stage, and $k_{2}\;\left(<k_{0}\right)$, above which the population is dominant in the final stage. A stationary state is approximately retained between them for $k_{1}+1\leq k\leq k_{2}$ in the most time during the approach to equilibrium (assumption of *quasi-stationary flow*). We then have

(106) $$J_{k}≃\left\{\begin{array}{cc} 0 & \left(1\leq k\leq k_{1}\right)\\ \mathrm{independent}\;\mathrm{of}\;k\equiv J & \left(k_{1}+1\leq k\leq k_{2}\right)\\ 0 & \left(k_{2}+1\leq k\right) \end{array}\right.$$

In Appendix C, we show that the flux *J* is proportional to the probability of free groups as

(107) $$RJ≃\frac{m_{0}+M_{1}}{M_{0}}\xi_{1}$$

where $m_{j}$ and $M_{j}$ are the *j*-th moments for small aggregates and large micelles defined by

(108a)mj≡∑k=1k1kjp¯k(108b)Mj≡∑k=k2+1∞kjp¯k

for j=0,1,2⋯. Here, the resistance *R* in the heat flow analogy is given by

(109) $$R\equiv \sum_{k=k_{1}+1}^{k_{2}}\frac{1}{\beta_{k}K_{k}{\bar{z}}^{k}}=\frac{1}{\psi }\sum_{k=k_{1}+1}^{k_{2}}\frac{k}{\beta_{k}{\bar{p}}_{k}}$$

We also find that the probability of large micelles for $k\geq k_{2}+1$ is approximately given by

(110) $$\xi_{k}≃k\xi_{1}-RJ$$

The kinetic equation for k=1 then takes the form

(111) $$\frac{d\xi_{1}}{dt}=-2J_{2}-\sum_{k=3}^{\infty }J_{k}≃-\frac{m_{1}+M_{1}}{RM_{0}}\left(k_{2}-k_{1}\right)\xi_{1}$$

Hence, the relaxation time is

(112) $$\frac{1}{t_{R}}=\left(k_{2}-k_{1}\right)\frac{m_{1}}{RM_{0}}\left(1+\frac{M_{1}}{m_{1}}\right)$$

which is similar to AW formula. The solution of (111) is given by

(113) $$\xi_{1}\left(t\right)=Ce^{-t/t_{R}}$$

with $C\equiv \left(1-{\bar{p}}_{1}\right)/{\bar{p}}_{1}$.

Substituting (110) into the gel-point condition (105), we have

(114) $$f^{\prime }\left\{\sum_{k=2}^{k_{1}}\left(k-1\right)k{\bar{p}}_{k}+\sum_{k\geq k_{2}+1}\left(k-1\right)k{\bar{p}}_{k}\right\}\xi_{1}\left(t_{g}\right)-f^{\prime }RJ\sum_{k\geq k_{2}+1}{\bar{p}}_{k}=1-f^{\prime }\sum_{k\geq 2}\left(k-1\right){\bar{p}}_{k}$$

and hence we finally find the form

(115) $$t_{g}=t_{R}Q\left(\bar{z}\right)$$

for the gelation time with the thermodynamic factor

(116) $$Q\left(\bar{z}\right)=\ln\left\{\frac{f^{\prime }CM}{f^{\prime }\sum_{k\geq 2}\left(k-1\right){\bar{p}}_{k}-1}\right\}$$

where

(117) $$M\equiv \frac{M_{1}}{M_{0}}\left(m_{1}+M_{1}\right)-m_{2}-M_{2}$$

**Figure 7:**
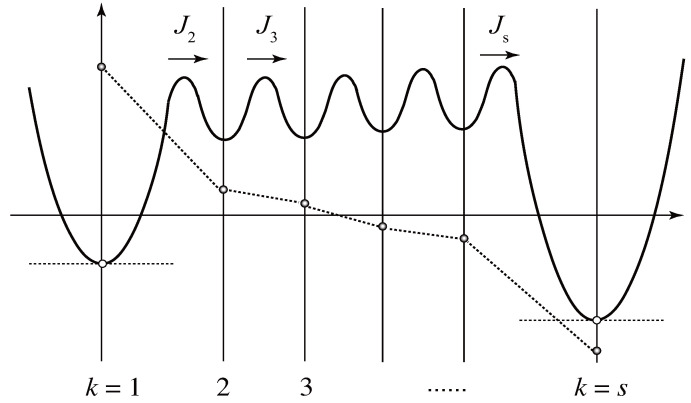
Quesi-stationary state of the stepwise cross-linking for the multiplicity with upper bound. The intermediate cross-link junctions (k=2,⋯,s−1) are close to equilibrium with $\xi_{k}≃0$, and hence the fluxes $J_{k}\;\left(k=2,\cdots ,s-1\right)$ are kept nearly constant.

To see the relaxation time more specifically, we consider the special case in which the multiplicity of the cross-links is limited from above such that only k=1,2,⋯s are allowed. Then, the last step from s−1 to *s* is expected to be slower than other steps, and hence we can choose $k_{1}=2$ and $k_{2}=s-1$. Figure 7 shows the quasi-stationary approximation schematically. Circles connected by dotted lines indicate the probability deviation $\xi_{k}\left(t\right)$ at time *t*. Since the moments are reduced to $m_{1}=m_{2}={\bar{p}}_{1}$ and $M_{0}={\bar{p}}_{s},M_{1}=s{\bar{p}}_{s}$, we have

(118) $$\frac{1}{t_{R}}=\left(s-2\right)\frac{{\bar{p}}_{1}}{R{\bar{p}}_{s}}\left(1+s\frac{{\bar{p}}_{s}}{{\bar{p}}_{1}}\right)$$

This is again KHDS form with an effective rate constant

(119) $$\beta_{\mathrm{eff}}=\left(s-2\right)\frac{{\bar{p}}_{1}}{R{\bar{p}}_{s}}$$

written in terms of the resistance constant

(120) $$R\equiv \sum_{k=2}^{s-1}\frac{k}{\beta_{k}{\bar{p}}_{k}}=\sum_{k=2}^{s-1}\frac{1}{\beta_{k}K_{k}{\bar{z}}^{k}}$$

Let us compare this with the relaxation time of the fixed multiplicity model (55) whose association takes place simultaneously. In the stepwise association, cross-link junctions grow one by one from smaller to larger until they are saturated. All intermediate states must reach equilibrium before the last one (largest junction), and hence they serve as a resistance for the solution to go back to equilibrium. As a result, the dissociation rate β is replaced by an effective one $\beta_{\mathrm{eff}}$. Relaxation is much slower, in particular, for the high concentration region.

**Figure 8:**
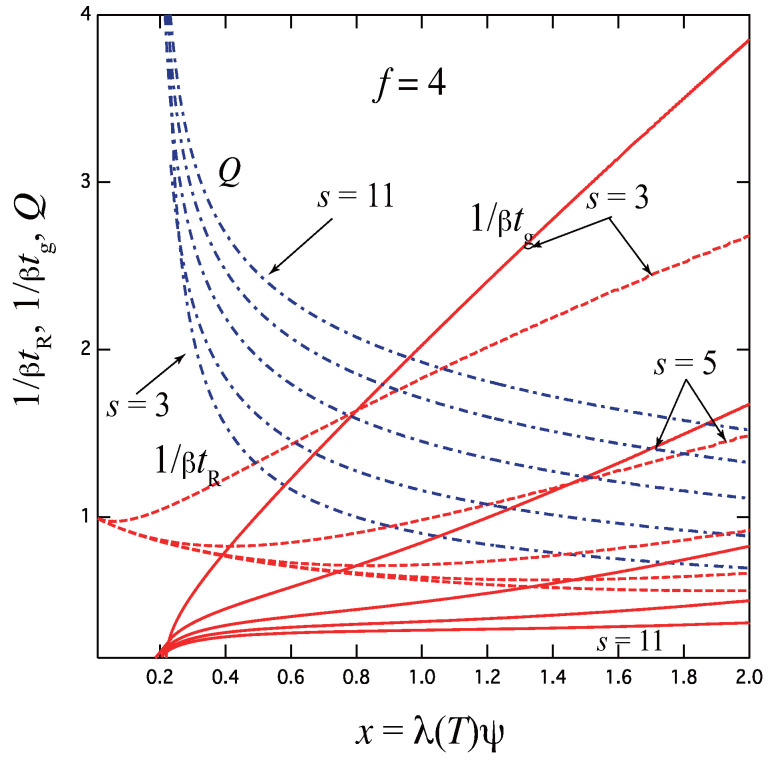
The reciprocal gelation time $1/\beta t_{g}$ (solid lines), relaxation time $1/\beta t_{R}$ (dotted lines) and the thermodynamic factor *Q* (broken dotted lines) plotted against the scaled concentration x≡λ(T)ϕ for the functionality f=4 with varied upper limit s=3,5,7,9,11 of the junction multiplicity. With increasing *s*, the gelation time becomes slower with a smaller slope at high concentration region.

Figure 8 shows the gelation time $t_{g}$, the relaxation time $t_{R}$, and the thermodynamic factor *Q* plotted against the scaled concentration x≡λ(T)ϕ for the functionality f=4 with the varied upper bound s=3,5,7,9,11 of the junction multiplicity. For simplicity, uniform $\beta_{k}$ is assumed. The case of s=3 goes back to the three-state model studied above. With an increase of the upper limit *s*, relaxation becomes slower because the resistance *R* due to the stepwise time development increases by the existence of many intermediate states. This tendency is opposite to the simultaneous formation of the fixed-multiplicity junctions by one step, for which relaxation becomes faster with *k*. In the high concentration region, the reciprocal relaxation time behaves with a power index 2/s as $1/t_{R}∼x^{2/s}$. We can study these results in more detail by comparing the experimental data of associating polymer solutions measured by using a temperature-jump technique.

## 4. Discussion

On the basis of the kinetic equation of cross-linking reaction, we have found the gelation time in the temperature/concentration jump experiments. In the high concentration region, we find the power law $t_{g}^{-1}∼x^{n}$ with the power index *n*: n=1 for pairwise cross-linking, n=k−1 for simultaneous association of *k* functional groups, and n=2/s for stepwise association with the maximum multiplicity *s*. These results can be compared with the experimental data for (1) to study the formation process of the cross-links. For the amplitude, we find

(121) $$A\left(T\right)≃{\lambda (T)}^{n}∼\exp\left(n\Delta H/k_{B}T\right)$$

where ΔH is the enthalpy of association. The factor n=k−1 is associated because there are k−1 bonds in a cross-link of multiplicity *k*.

In the vicinity of the gel point Δϕ<<1, on the other hand, we have found a logarithmic singularity n=0. The index corresponds to the critical exponent of a phase transition. It is generally a fractional number. However, since our kinetic equation is based on a mean-field picture (equal reactivity), we have reached a mean-field exponent n=0.

As for the temperature quench, we can do a similar analysis in the critical region by the plot

(122) $$t_{g}^{-1}=B\left(\phi \right){\left(\Delta T\right)}^{m}$$

We have again m=0 for the index. For the deep quench temperature jump experiment ($\Delta T/T_{\mathrm{gel}}>>1$), however, we exponential law (121).

For simplicity, we assumed cold-setting gelation, i.e., gelation at low temperature. In nature, however, we often observe heat-setting gelation phenomena. Some examples are biopolymer hydrogels and temperature-responsive polymers in water. Polymers are dissolved in water due to hydration. When the temperature is raised, polymers are dehydrated, and exposed to water, followed by the intermolecular association of the hydrophobic segments interfering with LCST phase separation. For heat-setting gelation, therefore, we have to consider the dehydration process (dissociation of bound water molecules from the polymer chain segments) before the cross-linking reaction. We will treat them in a separate paper.

Throughout this paper, we have confined our study to the pre-gel region $t\leq t_{g}$ before the solution reaches the gel point. In this region, the conservation law (6) of the functional groups, equilibrium reactivities ${\bar{p}}_{k}$ (5), and the gel-point condition (48) all hold without ambiguity as they are given in the text. From the equilibrium constants, we can derive the detailed balance condition for the rate constants in the kinetic equation. However, in the post-gel region $t_{g}\leq t$ where gel networks exist, there is a possibility such that the reaction within the gel part may be different from that of the sol part.

There are several treatments of the reaction in the post-gel region in the theoretical study of polycondensation by tree statistics; one assumes a tree structure for a gel network as for the sol, but the other permits cycle formation within the network. The former was proposed by Stockmayer [33], and the latter by Flory [41,42,43]. Later, Ziff and Stell [35,36,37,38,39] examined another possibility from a kinetic point of view. We have, however, avoided this problem because, for the estimation of the gelation time, we need only information on the reactivities before the gel point. After the gel point is passed, the solution, in particular its sol part, may change very differently depending on the mechanism of cross-linking reaction.

## 5. Conclusions

We have presented a very general theoretical framework for the study of the gelation time of thermoreversible gels with specified multiple structures of cross-link junctions. It is based on the kinetic equation for the stepwise association of functional groups. All results are presented from a unified point of view in terms of the *gelation-time diagram*—simultaneous plot of $1/t_{g},1/t_{R}$ and *Q* against the scaled concentration variable x≡λ(T)ψ in a single graph.

From the theoretical modeling, the following conclusions can be drawn:

(1) The gelation time $t_{g}$, the relaxation time $t_{R}$, and the thermodynamic factor *Q* are all functions of a single variable x≡λ(T)ψ (scaled concentration), where λ(T) is the stepwise association constant at the final temperature *T* at which cross-linking reaction proceeds. Therefore, temperature and concentration are not separable, but give the same effect if they are properly scaled under a fixed value of *x*. Data for different concentrations can be superimposed onto a single curve by using an appropriate temperature shift factor.
(2) These three factors obey a fundamental relation $t_{g}\left(x\right)=t_{R}\left(x\right)Q\left(x\right)$. The thermodynamic factor Q(x) is logarithmically singular at the equilibrium gel point $x=x_{g}$, while the relaxation time $t_{R}\left(x\right)$ is continuous across the gel point. They are calculated for some important models of cross-link junctions, such as pairwise cross-linking, three-state model, cross-linking with fixed high multiplicity, and micellar cross-linking.
(3) The gelation time $t_{g}\left(x\right)$ of reversible cross-linking approaches the power law of the irreversible one in the asymptotic region of large *x* (either high concentration ϕ or high values of the association constant λ(T)). The power index of $1/t_{g}$ lies at somewhere between k−1 (simultaneous cross-linking) and 2/s (stepwise cross-linking). Hence, the reaction kinetics, simultaneous or stepwise, can be inferred by measuring the power.
(4) For large micellar cross-link junctions, the gelation time is derived on the basis of the quasi-stationary approximation (Aniansson–Wall formula) for the relaxation time. Combination with the singular part of the thermodynamic factor estimated by our preceding equilibrium gelation theory provides an accurate estimation of the gelation time, and enables a comparison with experimental data.

The model solutions proposed in this study have obvious advantages in finding the microscopic parameters regarding the cross-linking reaction, such as stepwise rate constants and cross-link multiplicity, from macroscopic measurements on the gelation time and the relaxation time.

Our theoretical framework may directly be applicable to some important thermoreversible gels for which the equilibrium sol–gel transition lines are established. We hope detailed experimental data on the gelation time for the systems treated here will be reported in the near future.

## Appendix A. Solution of the Kinetic Equation

Because of the second order nature of the reaction, we have a factorized form

(A1) $$g\left(p\right)=\left(p-\eta^{\left(+\right)}\right)\left(p-\eta^{\left(-\right)}\right)$$

with Equation (15). Equation (11) can easily be integrated. The solution is given by

(A2) $$p\left(\tau \right)=\frac{\left[\eta^{\left(+\right)}-p\left(0\right)\right]\eta^{\left(-\right)}-\left[\eta^{\left(-\right)}-p\left(0\right)\right]\eta^{\left(+\right)}e^{-\gamma \tau }}{\left[\eta^{\left(+\right)}-p\left(0\right)\right]-\left[\eta^{\left(-\right)}-p\left(0\right)\right]e^{-\gamma \tau }}$$

where $\gamma \equiv \eta^{\left(+\right)}-\eta^{\left(-\right)}$ is the relaxation rate, and p(0) is the initial value of the reactivity. We assume for simplicity that the initial temperature is sufficiently high, so that all functional groups are free p(0)=0. Hence, we have the result (14).

## Appendix B. Retardation Coefficient

To find the retardation coefficient and investigate the behavior of the gelation time in a high concentration region, we consider the integral of the Equation (42)

(A3) $$\tau =\int_{0}^{p}\frac{dp}{{\left(1-p\right)}^{k}-\beta^{\prime }p}$$

where $\beta^{\prime }=1/kx^{k^{\prime }}$. Expanding the integrand in powers of $\beta^{\prime }$, and fixing the reactivity at its gel-point value $p_{g}=1/f^{\prime }k^{\prime }$, we have

(A4) $$\tau_{g}=\sum_{m=0}^{\infty }I_{m}\left(p_{g}\right){\beta^{\prime }}^{m}$$

where integrals are defined by

(A5) $$I_{m}\left(p_{g}\right)\equiv \int_{0}^{p_{g}}\frac{p^{m}dp}{{\left(1-p\right)}^{k(m+1)}}$$

Then, the gelation time takes the form

(A6) $$\beta t_{g}=\frac{I_{0}\left(p_{g}\right)}{kx^{k^{\prime }}}\left\{1+\frac{R_{f,k}}{x^{k^{\prime }}}+O\left(\frac{1}{x^{2k^{\prime }}}\right)\right\}$$

and hence we have

(A7) $$R_{f,k}=I_{1}\left(p_{g}\right)/kI_{0}\left(p_{g}\right)$$

for the retardation coefficient. We have already found it in (29) for the pairwise cross-linking k=2. For a general *k*, it is explicitly given by Equation (64). The first order correction to gelation time is proportional to $1/x^{k-1}$.

## Appendix C. Quasi-Stationary Approximation

Because the flux $J_{k}$ is given by

(A8) $$J_{k}≃\beta_{k}K_{k}{\bar{z}}^{k}\left(\xi_{k-1}-\xi_{k}+\xi_{1}\right)\;\mathrm{for}\;k\geq 2$$

in the linear approximation, we find for $k\geq k_{2}+1$

(A9) $$\sum_{k=2}^{k}\left(\xi_{k-1}-\xi_{k}+\xi_{1}\right)=\sum_{k=2}^{k}\frac{J_{k}}{\beta_{k}K_{k}{\bar{z}}^{k}}≃J\sum_{k=k_{1}+1}^{k_{2}}\frac{1}{\beta_{k}K_{k}{\bar{z}}^{k}}$$

The left hand side is simply $-\xi_{k}+k\xi_{1}$ by cancellation, so that we have

(A10) $$\xi_{k}≃k\xi_{1}-RJ$$

where *R* defined by (109) is the analogy of the resistance in the heat flow. Similarly, for $k\leq k_{1}$, we find

(A11) $$\xi_{k}≃k\xi_{1}$$

To find *J*, we substitute these relations into the materials conservation law $\sum p_{k}=1$. We find

(A12) $$\sum_{k=1}^{k_{1}}{\bar{p}}_{k}\left(k\xi_{1}\right)+\sum_{k=k_{1}+1}^{\infty }{\bar{p}}_{k}\left(k\xi_{1}-RJ\right)≃0$$

and hence we have (107).
